# Pacing behaviour of players in team sports: Influence of match status manipulation and task duration knowledge

**DOI:** 10.1371/journal.pone.0192399

**Published:** 2018-02-05

**Authors:** Ricardo Ferraz, Bruno Gonçalves, Diogo Coutinho, Daniel A. Marinho, Jaime Sampaio, Mário C. Marques

**Affiliations:** 1 Department of Sports Sciences, University of Beira Interior, Covilhã, Portugal; 2 Research Center in Sports Sciences, Health Sciences and Human Development, CIDESD, Vila Real, Portugal; 3 Department of Sports Sciences, University of Trás-os-Montes and Alto Douro, CreativeLab Research Community, Vila Real, Portugal; Universita degli Studi di Verona, ITALY

## Abstract

The study aimed to identify the influence of prior knowledge of exercise duration associated with initial information about momentary match status (losing or winning) on the pacing behaviour displayed during soccer game-based activities. Twenty semi-professional male players participated in four game scenarios divided in two sessions. In the first game scenario, players were not informed about the time duration or initial match status. In the second, players were only informed they would be required to play a small-sided game for 12 minutes. In the third, players were told they would play a small-sided game for 12 minutes and that one of the teams was winning 2 to 0. Finally, in the fourth game scenario, players were instructed they would play a small-sided game for 12 minutes and the score lines used at the start of the previous game scenario were reversed. The results showed a tendency for the unknown task duration to elicit greater physical responses in all studied variables, compared with knowing the task duration. Knowing the task duration and starting the game winning or losing did not affect the players’ activity profile between the two conditions. Thus, during small-sided soccer games, knowledge (or not) about the exercise duration alters the pacing behaviour of the players. Moreover, short and undisclosed-length exercise durations resulted in the adoption of more aggressive pacing strategies, characterised by higher initial exercise intensities. Furthermore, previous information on match status does not seem to interfere with pacing patterns if the players are aware of the exercise duration. Coaches may use knowledge of exercise duration to manipulate the small-sided games’ demands.

## Introduction

The concept of pacing behaviour has become increasingly important in continuous sports, such as cycling or running, and intermittent team sports, such as soccer [1–7]. During self-paced exercise, a pacing strategy is dependent on the environment, contextual factors, exercise demands goals, and afferent physiological feedback [8]. In soccer, pacing can be defined as the distribution of energy resources that optimise match running performance [5]. The distribution suggests that a voluntary or involuntary behaviour (effort) may limit performance rather than the absolute capacity of a single physiological system [9].

Until recently, match running performance in soccer was thought to be the result of physiological and psychological factors, such as blood lactate and H^+^ accumulation, glycogen depletion, PCr depletion, dehydration, intramuscular acidosis, insufficient Ca^2+^ within muscles, neural transmission failure, motivational and mental mechanisms, or contextual and tactical factors [5, 10, 11]. However, according to recent psychophysiological theoretical approaches, match running performance in soccer is not only the result of the classic theory of fatigue [12] but also due to the player’s ability to manage the effort, consciously or not, to complete the activity in a reasonable physiological state, by controlling the onset of fatigue [2, 13]. Sensations may be sent from the musculoskeletal and cardiovascular systems to the central nervous system (called afferent sensations), where the pacing pattern is altered based on the brain’s interpretation of the perceived exertion [2, 13]. During the game and based on the affordance competition hypothesis (AC-hypothesis), pacing could be considered an interactive phenomenon at a neuropsychological level and a behavioural expression of decision making based on perception of affordances [14, 15]. Between competing athletes of comparable physical capacities, the ability to manage effort during exercise and its resulting decision-making aspect might be decisive in the performance and result of the game [4, 15, 16].

To optimise running performance in soccer, whole-match players adopt a slow positive pacing profile, characterised by a gradual decline in total and high-intensity running, whereas part-match players select either ‘all out’ or ‘reserve’ strategies [5, 10]. The few experimental studies available suggest that knowledge of the exercise duration justifies the athlete’s pacing in soccer play. This effect acts as a regulator of exercise performance and is commonly approached throughout deception-based studies (i.e. participants believe they are exercising for a given time period but are asked to continue exercising longer towards the completion of this time). The increase in rate of perceived exertion (RPE) when the deception is revealed leads to the slowing of the pace without physiological reasons and may reflect a disruption of the feed forward/feedback mechanism. A number of studies have reported processes by which athletes can hold back a security physiological reserve during exercise of an uncertain duration [17–19].

During a soccer game or even during training exercises, players’ pacing behaviour may, in part, be anticipated based on knowledge of the exercise duration and possibly other contextual variables that can play a decisive role in the regulation of effort, such as match status. Indeed, these factors have recently been identified as important aspects in the physical and physiological effects of pacing behaviour [1]. Thus, during soccer activities, the decision to reduce, increase, or maintain a given power output will depend on the perceived benefits of each moment, based on the idea that when the perceived ‘payoff’ is potentially large (e.g. to be wining and to know the exercise duration) or when it is necessary to achieve an objective (e.g. to be losing and having to win or draw, in reference to the remaining playing time), an athlete would be more likely to incur a greater degree of homeostasis disruption, the medical term for the unstable condition of an organism and of its internal environment [8].

Available research in team sports suggests that players alter their pacing behaviour based on their knowledge of the exercise duration. Ferraz et al. [1] stated that during soccer in small-side games (SSG) knowledge of having to exercise for a short duration leads to an increase in exercise intensity. Furthermore, longer exercise durations may decrease differences in physical performance between having full and no knowledge of the exercise duration. Although conclusions on longer exercise duration are better supported, the conclusions regarding short-term exercise as well as the role of time duration factor need to be clarified. Gabbett et al. [2] found a significant effect of condition and time duration factor on the relative intensity of SSG with higher initial intensities, in the context of rugby, but the precise role of knowledge of task duration remained unclear.

Regarding the importance of match status, the available results are not consistent. Gabbett [20] suggested that physical effort is higher when winning compared with losing. However, Sampaio et al. [21] did not find significant differences in heart rate (HR) according to the final score. Di Salvo et al. [22] reported that less successful teams cover more distance than their winning counterparts. Sullivan et al. [23] found that in a less-successful team, players demonstrate increased physical activity profiles and decreased skill involvement and proficiency. These studies analysed exercise performance considering the final performance between the team that lost and the team that won. No studies have attempted, from a psychophysiological perspective, to analyse the influence of the match status (to start losing or winning) on players’ pacing behaviour based on previous information provided (in anticipation). Indications of varied pacing strategies are observed in team sports, possibly indicating that winning teams may regulate their efforts allowing for an end-spurt of high-intensity activity [24]. Therefore, this study aimed to identify the influence of prior knowledge of exercise duration combined with initial match status information (such as having an advantage or disadvantage) on the pacing patterns employed during game-based exercise activities in soccer. We hypothesise that players alter their pacing pattern based on knowledge of the exercise duration and according to the initial match status (to start losing or winning).

## Methods

### Participants

Twenty semi-professional male soccer players (mean ± SD: age 21.9 ± 2.1 years; body height 1.80 ± 0.06 m; body mass 75.7 ± 5.8 kg) with 9.1 ± 3.8 years of experience, playing in the second division of the Portuguese National Competition, participated in this study. All players performed five training sessions per week (90 min each) and played one official game during the weekend. All participants were fully informed about the protocol before participating in this study. An informed and written consent was obtained prior to testing from all participants, in accordance with the approval by the Ethics Committee of the Research Centre for Sport Sciences, Health and Human Development, based at Vila Real (Portugal) and with the principles of the Declaration of Helsinki.

### Design

A cross-sectional field study and an adaptation of the Ferraz et al. [1] protocol were used. The protocol involved testing conducted over two training sessions during competitive season. The first session involved familiarisation of players with the game-based activity, equipment, and procedures. Within one week of the familiarisation session, the players performed four randomised SSG divided in two sessions and separated by seven days. In the first game scenario, players were not informed about the time duration or initial match status. In the second, players were only informed they would be required to play the SSG for 12 minutes. In the third, players were told that they would play the SSG for 12 minutes and that one of the teams started the SSG winning 2 to 0. Finally, in the fourth game scenario, players were instructed that they would play the SSG for 12 minutes and the team that in the previous game started to win, now starts to lose 2 to 0. To best control for circadian variations on the measured variables, all games were performed at the same time during the day (from to 17:00 pm until 19:00 pm), and during these sessions, the average temperature recorded was 20°C.

### Methodology

Two SSGs were played in each training session. Players were divided into four balanced teams of five players, taking into account playing positions, tactical/technical levels, and physical capacities [25]. Teams’ constitutions and respective opponents were maintained during all conditions. The aim of the game was to outscore the opponents. The games were played on a standardised (20 x 40 m) pitch dimension of 80 m^2^ per player with no goalkeepers present in each team. The goal size during these games were also standardised at 2.5 x 1.5 m. Multiple balls were positioned around the pitch; in the case of a ball leaving the pitch, another was quickly introduced to ensure game continuity. No coach feedback or encouragement was allowed during the games [26].

At the completion of each game, the players provided an overall rating of their perceived exertion using the Borg 6–20 scale (RPE) [27]. All players were familiar with the RPE scale and had previous experience in rating their perceived exertion of training drills and SSG. Blood lactate concentrations were measured after each SSG. Blood was taken from the fingertip, whereas lactate measurement was performed using a portable machine (Roche Accutrend Lactate Test Strips, Basel, Switzerland).

Players’ tracking displacements were recorded by global positioning system units working at a sampling frequency of 5 Hz (SPI-Pro X II, GPSports, Canberra, ACT, Australia) [28]. The processed variables were the total distance covered (m), game pace (obtained by the players’ average speed displacements), and total body impacts [29]. In addition, three ratios were calculated to relate and compare the distance covered at high to very high (above 16 km/h), moderate (10.0–15.9 km/h), and low (7.0–9.9 km/h) intensities, with distance covered at very low intensities (0–6.9 km/h) normalised for each 100 m for comparison purposes. These work–rest ratios are frequently used to describe activity profiles [30].

### Statistical analysis

A descriptive analysis was performed using mean and standard deviations. Magnitude-based inferences and precision of estimation were employed [31]. Comparisons among game conditions were assessed via standardised mean differences with 90% confidence intervals [32, 33]. Thresholds for effect size (ES) statistics were 0.2, for trivial; 0.6, for small; 1.2, for moderate; 2.0, for large; and 2.0, for very large [33]. Differences in means were estimated in per cent units through log-transformation to reduce the non-uniformity of error; uncertainty in the estimate was expressed as 90% confidence limits (CL). The smallest worthwhile differences were estimated from the standardised units multiplied by 0.2. Probabilities were used to make a qualitative mechanistic inference on the true effect (i.e. if the probability of the effect being substantially higher and lower was > 5%, the effect was reported as unclear). Otherwise, the effect was clear and reported as the magnitude of the observed value. The scale was as follows: 25%−75%, possible; 75%−95%, likely; 95%−99%, very likely; > 99%, almost certain [33]. The intra-day variability (between-players) in RPE and blood lactate variables was measured as typical error for each session and expressed as a coefficient of variation, CV (%) [34].

## Results

### Session characterisation

**Fig 1:**
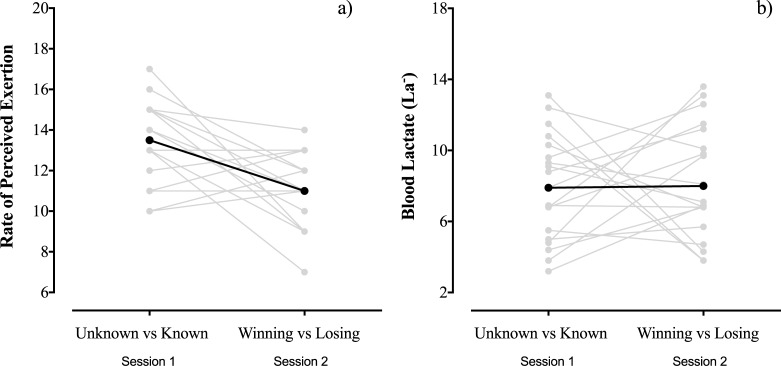
Characterisation of rate of perceived exertion and La^-^ according to each testing session (Session 1: Unknowing and knowing the task duration; Session 2: Knowing the task duration and winning or losing). Note: Grey solid lines indicate the responses of individual participants; black dotted lines indicate the group mean value.

The characterisation of each data collection session on RPE and La- levels is presented, respectively, in Fig 1A and 1B. The players’ RPE scores were higher when knowledge of the task duration was manipulated but did not differ between winning and losing conditions (means variation %; ± 90 confidence limits, 18.7%; ± 7.6%, large effect). In contrast, the La- values showed unclear differences between the two sessions. The intra-day measures (between-players) presented 14.2% variation (coefficient of variation) in RPE when knowledge of the task duration was manipulated and 15.3% variation in the winning and losing conditions. Meanwhile, the La- measures presented 37.8% variation when knowledge of the task duration was manipulated and 38.6% variation in winning and losing conditions.

### Physical performance

**Fig 2:**
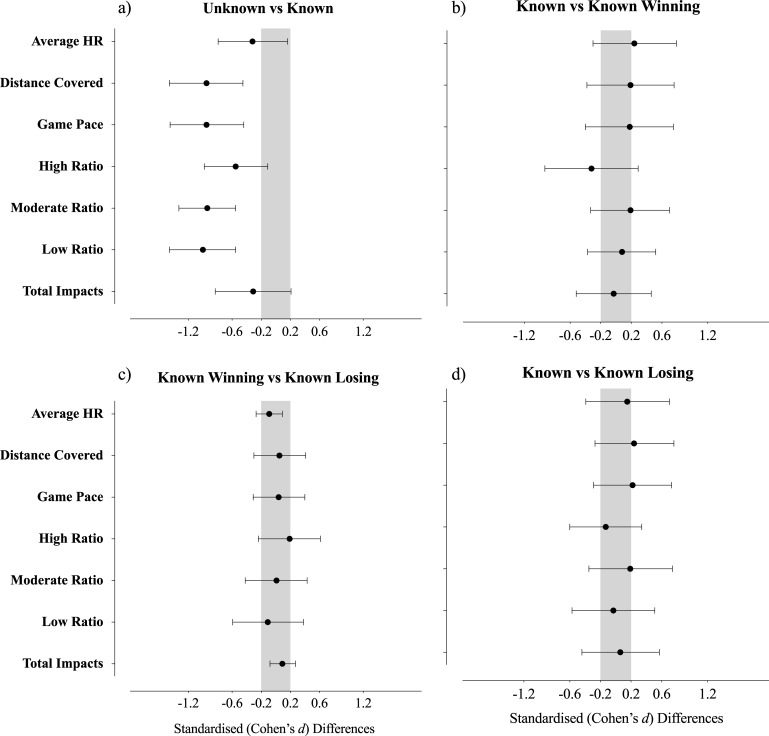
Standardised (Cohen) differences in physical variables according to (a) Not knowing vs knowing the task duration; (b) Know the task duration and start winning vs Know the task duration and start losing; c) Know the task duration vs Know the task duration and start winning; d) Know the task duration vs Know the task duration and start losing. Error bars indicate uncertainty in the true mean changes with 90% confidence intervals.

The effects of the initial task condition on the players’ physical performance are presented in Table 1 and Fig 2. The unknown task duration (see Fig 2A) elicited greater physical responses in all studied variables, compared with knowing the task duration. Not knowing the task duration presented very likely 11% higher in total distance covered, full stop (moderate effect, standardised differences; ± 90% confidence limits, −1.0; ± 0.5). Additionally, a very likely 11% higher game pace (moderate effect, −1.0; ± 0.5), most likely 26% higher moderate ratio (moderate effect, −0.9; ± 0.4), and most likely 8% higher low ratio (moderate effect, −1.0; ± 0.5) were recorded. There was a likely 29% higher values high ratio when there was no information on the task duration than when there was information about task duration (moderate effect, −0.6; ± 0.4).

**Table 1:** Descriptive statistics on the different condition variables.

Variables	Unknown	Known	Known Winning	Known Losing	Change in mean (%; ±90% confidence limits) Uncertainly in true differences, Non-clinical inference			
					a	b	c	d
Total distance meters/min	1073.1±87.6	961.6±131.0	981.4±118.3	987.0±110.2	-10.9; ±5.4, very likely–ive	2.3; ±7.1, unclear	3.0; ±6.4, unclear	0.6; ±4.3, unclear
Ratio (H/VH) meters	26.6±9.8	20.0±9.4	17.8±10.6	19.2±10.2	-29.1; ±19.8, likely–ive	-18.2; ±32.1, unclear	-8.0; ±27.6, unclear	12.5; ±30.3, unclear
Ratio (moderate) meters	78.7±21.9	59.0±20.0	63.1±23.5	63.1±20.5	-26.2; ±9.4, most likely–ive	6.3; ±17.7, unclear	6.5; ±18.9, unclear	0.2; ±13.7, unclear
Ratio (low) meters	154.5±13.0	141.4±8.1	142.7±12.4	141.5±14.4	-8.3; ±3.6, most likely–ive	0.7; ±3.8, unclear	-0.3; ±4.6, unclear	-0.9; ±4.1, unclear
Total impacts a.u.	190.5±97.0	156.8±74.2	152.0±70.3	158.1±77.7	-16.1; ±24.9, unclear	-1.6; ±27.8, unclear	3.7; ±30.3, unclear	5.5; ±10.5, likely trivial
Game pace km/h	6.4±0.5	5.8±0.8	5.9±0.7	5.9±0.7	-10.8; ±5.4, very likely–ive	2.2; ±7.1, unclear	2.7; ±6.4, unclear	0.5; ±4.3, unclear
Avg HR a.u.	174.1±13.1	168.8±14.9	173.3±18.0	171.8±18.4	-3.2; ±4.7, possibly–ive	2.5; ±5.7, unclear	1.6; ±5.6, unclear	-0.9; ±1.8, likely trivial

**Note:** Abbreviations and symbols: -ive = negative. Differences in means (%; ± 90% CL) are identified as: a) Unknown vs. Known; b) Known vs. Known Winning; c) Known vs. Known Losing; d) Known Winning vs. Known Losing

Fig 2B presents the ES with mean differences for unclear effects between winning/losing across knowing task duration scenarios. Knowing the task duration and starting the game winning or losing presented unclear effects on players’ activity profile when comparing the two conditions. Fig 2C and 2D present the results on the effects of knowing the task duration and the combined effect of knowing the task duration and starting winning (2a) or starting losing (2d).

## Discussion

This study aimed to identify the influence of prior knowledge of exercise duration associated with initial information about match status (starting from a losing or winning position) on pacing behaviour during soccer SSG. The main findings indicated that the manipulation of the knowledge of task duration influences players’ physical performance during the exercise, whereas starting the exercise winning or losing does not show effects on players’ performance.

Our findings are consistent with previous literature reporting that during soccer SSG, players alter their pacing strategies based on their knowledge of the exercise duration [1]. Moreover, these results reinforce a tendency already demonstrated in cyclical sports and experimental closed approaches [2, 35–40]. In rugby, players managed their pacing strategies based on the anticipated endpoint of the exercise bout [2, 3]. These findings also provide support for the key role of the central nervous system in the regulation of exercise performance. Indeed, prior knowledge of the exercise duration may be important information that the brain uses to select and adopt the appropriate exercise intensity. In this sense, the brain may initiate a self-regulation strategy of effort based on prior knowledge of the task, which leads to changes in performance on the basis of previous information provided [1]. The decision to reduce or increase efferent neural drive, manifested in a decrease or increase in physical performance, is based on the degree of uncertainty or certainty about the exercise duration and the degree of potential homeostasis disruption perceived in each condition, in connection with afferent feedback and feed forward systems [8]. Previous deception studies [17–19] revealed that responses to exercise without knowledge of duration may reflect subconscious improvement in exercise economy to conserve energy owing to the unknown duration of the exercise bout. However, in the current study, and contrary to most deception studies, the unknown condition resulted in higher exercise intensity and perceived exertion, which can be attributed to the time duration factor and the lack of knowledge about it.

One important point that this study highlights is the importance of the exercise duration connected with the information about this time. Previously, Ferraz et al. [1] proposed that a longer exercise duration, even with full knowledge of this duration, could lead to greater uncertainty in effort regulation and an increase in exercise economy. The pacing pattern between the unknown and known condition therefore tends to be similar because the degree of uncertainty in both conditions may be similar. Consequently, they concluded that no differences in pacing patterns exist between having or not having knowledge of exercise duration, with respect to the long-term duration of exercise in SSG. The current study identified a tendency that most of the variables present higher values during the unknown exercise duration scenario, based on short-term exercise duration. This trend is supported by the studies of Gabbett et al. [2], Highton et al. [3] and Ferraz et al. [1]; in these studies, most of the physical variables showed a tendency to be higher in the first few minutes of the exercise, which was also verified previously in sprinting analysis [35].

This regulation in the pacing behaviour demonstrates the tendency of players, during SSG, to begin the exercise with a higher intensity compared with the rest of the exercise duration. This behaviour is reinforced if the player does not know the duration of the session, although recent suggestions on this topic indicate otherwise [8, 41]. Nonetheless, the lack of knowledge of the duration of the task can be traced as the cause of such energy burst. Absent information on the duration of exercise, the brain does not have sufficient data to process the needed response, immediately going for an ‘all out’ initial pacing strategy [1, 8]. Conversely, information enables the brain to adapt, using a more balanced strategy from the point of view of regulation of effort that is being altered and adjusted over the exercise time in response to available afferent signals.

Accordingly, the existence of a critical time from which the CNS, through the sum afferent signals, is able to discern the degree of effort it is willing to expend for certain durations, seems possible [42, 43]. When the duration of the exercise is long, the regulation may incline towards being more economical and balanced [1] because the brain has more time to discern any effects and trends towards preserving a security physiological reserve. However, if the duration of the exercise is short and unknown, the effort that the body is willing to provide seems always to be maximal, with the brain selecting a more intense pacing strategy [5]. Our results support the anticipatory feedback and psychobiological theories, as well as reveal new data on this topic in team sports. Moreover, they confirm that during SSG, the brain has a decisive role in the regulation of effort in anticipation of the exercise duration [1, 2, 35].

The current results also suggest no influence from the initial information on match status (to start losing or winning) on the players’ performance. A recent study in a rugby league suggested that physical demands are greater when winning compared with losing, and the distance covered in all evaluated variables was higher for winning teams in contrast to losing teams [20]. In Australian football matches, players showed an increased physical activity profile when the team is less successful [24]. Malone and Collins [44], in a study based on SSG (hurling players), concluded that team success during SSG affected high-speed performance, with winning teams covering significantly more distance and spending more time at higher percentages. In contrast, Sampaio et al. [21] studied SSG in terms of numerical inequality (4 x 5 and 5 x 4) and suggested that winning teams cover less total distance owing to increased technical performance. Sampaio et al. [21] reported that less successful teams cover more distance than their winning counterparts. Our results suggest that starting the game with knowledge of winning or losing, even with knowledge of the shortness of exercise duration, does not influence the players’ performance. In addition, there was no effect of this complementary information (winning or losing) relative to the initial condition (knowing the duration).

According to central regulation of exercise performance and anticipatory feedback theory, players modulate their efforts in anticipation and use pacing behaviour based on previous exercise information. Some studies referred to variations in pacing behaviour as being based on information and contextual circumstances during the game (temperature or match status) [13]. Therefore, previous information on the match status, reinforced by the knowledge that the exercise has a short duration, would enable prediction of changes in the context of the match, and consequently, in players’ pacing behaviour. However, no effect was identified in the present work, which leads us to suggest that it is important to analyse the circumstances and context of the game scenarios. Indeed, the effort modulation of players during exercise is dynamic and takes into account not only prior information but also dynamic considerations during the exercise. The number of goals with which the teams start the game, either at an advantage or disadvantage, dynamics and alteration of the score game, time duration of the exercise, number of goals during the game, or time in which they happened may influence the analysis. Furthermore, playing in a training context or in a real game context would influence the importance of the score. Similarly, reaction to the score, either in training or in competition, will depend on each player’s ability, individual interpretation, and approach for surpassing each scenario. These psychological aspects should also be considered from the psychophysiological integrated perspective.

The RPE values of our study should also be carefully interpreted. The anticipatory feedback model proposes that RPE may reflect the available fuel sources during exercise and may be used to assist athletes to select the most appropriate pacing strategy [12]. According to the psychobiological model of Marcora [42], Marcora [43], RPE is a key determinant in the regulation of exercise performance; conscious regulation of pace is primarily determined by the effort perceived by the athlete. However, Ferraz et al. [1] highlighted that RPE may be a psychological and volatile construct that is altered by changes and the cognitive focus of the athlete [41, 45]. Renfree et al. [8], in response to this, presented substantial evidence that conscious RPE can be dissociated from physiological processes through a variety of purely psychological mechanisms. Thus, RPE cannot be considered an encompassing measure [46]; it could be an oversimplification of the psychophysiological construct and an insufficient measure to capture the wide range of sensations experience [8], especially in team sports such as a soccer or in activities related to SSG. Future investigations should provide evidence to clarify the role of RPE as a regulator and/or quantifier measure. For a complementary analysis, it will also be pertinent to observe the variation of RPE throughout entire exercise sessions and not only at the end.

Regarding blood lactate levels, the similar results presented at the end of the two sessions support conclusions from other studies: lactate values in SSG may be incompatible with the dynamic character of the exercise [47]. These values may be unable to provide accurate and immediate information on physical impacts, as collected data would reflect only the last few minutes of effort and not be representative of the exercise intensity. However, blood lactate results may also support the anticipatory exercise model. First, and based on the different sessions’ results, these values suggest that purely peripheral models of fatigue are unable to explain the non-linearity of the fatigue effect. Second, the fact that the lactate values reflect the effort of the final moments of each session proves that the differences between the sessions were in the initial minutes, where the effect of the lack of knowledge can be assumed to be greater. In this sense, despite the lactate values not predicting the full extent of the exercise effort, they provide experimental evidence that supports the hypothesised impact on pacing behaviour in this study.

## Conclusions

During SSG in soccer, knowledge of the exercise duration alters players’ pacing behaviour. Short exercise duration and absence of knowledge of the duration contribute to more intense pacing strategies. Meanwhile, previous information on the match status is not shown to interfere with pacing patterns if the players know the exercise duration. Coaches should consider this information for their training sessions, as well as the possibilities of their manipulation in terms of physical impact regulation during training exercises.

### Practical applications

This study confirms the importance of the knowledge of exercise duration as a variable in pacing patterns in soccer, and therefore, in the physical performance of players. Information on exercise duration is shown to be a great tool for controlling and manipulating training sessions, especially exercises such as SSG. Coaches can manipulate this variable to manage the fatigue effect in exercise. This point can be an important consideration in terms of the methodology of soccer training, specifically in the preparation and monitoring of training tasks. The best way for the coach to promote greater physical impact on SSG is to plan short-duration exercises or shorter blocks of the same (until 12 minutes) and not inform players about the duration. However, if the coach wants the player to have a more balanced performance from the physical point of view, he/she must inform the player about the duration and can also refer to the score of the game. To develop technical and tactical content in the context of lower physical impact and better ability to regulate effort, the coach can disclose the duration of the exercise. However, if the intent is to develop such content intensively from a physical point of view, this information should be withheld, and instead, reduced durations of SSG should be used (e.g. shorter duration blocks until 12 min).
